# Functional and Genomic Analyses of Alpha-Solenoid Proteins

**DOI:** 10.1371/journal.pone.0079894

**Published:** 2013-11-21

**Authors:** David Fournier, Gareth A. Palidwor, Sergey Shcherbinin, Angelika Szengel, Martin H. Schaefer, Carol Perez-Iratxeta, Miguel A. Andrade-Navarro

**Affiliations:** 1 Max-Delbrück Center for Molecular Medicine, Berlin, Germany; 2 Ottawa Hospital Research Institute, Ottawa, Ontario, Canada; 3 The University of British Columbia, Vancouver, British Columbia, Canada; Universita' di Padova, Italy

## Abstract

Alpha-solenoids are flexible protein structural domains formed by ensembles of alpha-helical repeats (Armadillo and HEAT repeats among others). While homology can be used to detect many of these repeats, some alpha-solenoids have very little sequence homology to proteins of known structure and we expect that many remain undetected. We previously developed a method for detection of alpha-helical repeats based on a neural network trained on a dataset of protein structures. Here we improved the detection algorithm and updated the training dataset using recently solved structures of alpha-solenoids. Unexpectedly, we identified occurrences of alpha-solenoids in solved protein structures that escaped attention, for example within the core of the catalytic subunit of PI3KC. Our results expand the current set of known alpha-solenoids. Application of our tool to the protein universe allowed us to detect their significant enrichment in proteins interacting with many proteins, confirming that alpha-solenoids are generally involved in protein-protein interactions. We then studied the taxonomic distribution of alpha-solenoids to discuss an evolutionary scenario for the emergence of this type of domain, speculating that alpha-solenoids have emerged in multiple taxa in independent events by convergent evolution. We observe a higher rate of alpha-solenoids in eukaryotic genomes and in some prokaryotic families, such as Cyanobacteria and Planctomycetes, which could be associated to increased cellular complexity. The method is available at http://cbdm.mdc-berlin.de/~ard2/.

## Introduction

**Figure 1:**
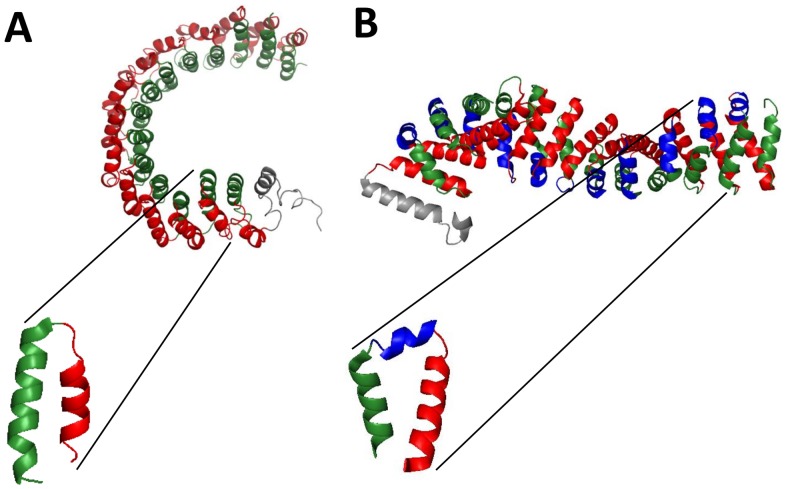
General features of alpha-solenoids. Alpha-solenoids are ensembles of repetitive units that assemble in an elongated and flexible domain. The repeats are formed by two anti-parallel alpha-helices. (A) The structure of the protein phosphatase 2A (PDB ID 2IAE chain A [8]) is an alpha-solenoid made of HEAT repeats (its two helices represented in green and red, respectively). (B) Armadillo repeats also form alpha-solenoids but have a small helix (in blue) between the two anti-parallel alpha-helices (structure of Beta-Catenin, PDB ID 2Z6H [60]).

Alpha-solenoids are elongated protein domains composed of repeated pairs of anti-parallel alpha-helices (Figure 1) [1]. The repeated units can be identified as sequence repeats (HEAT [2], Figure 1A and Armadillo [3], Figure 1B, among others) but there are examples where the sequence similarity between the repeated units is undetectable, hinting that structurally similar alpha-solenoids can be attained by convergent evolution. Alpha-solenoid repeats are very flexible [4], [5] and can be elastically extended and refolded when subjected to a mechanical stretch force [6]. This exceptional property renders them very efficient for protein-protein interaction (PPI) [7]. For example, the regulatory subunit B of the protein phosphatase 2A (coded by the *PPP2R5C* gene) is a known alpha-solenoid whose elastic deformations influence the opening and closing of the binding site for target proteins in the catalytic subunit C [8].

Detection of alpha-solenoids from protein sequence alone is relevant because such a prediction allows defining the secondary and tertiary structure of a large part of a protein with relatively high precision. For example, this has been used for the study of proteins of medical interest for which no solved 3D structures are yet available such as Huntingtin, whose mutation is responsible of Huntington's disease [9], or mTOR whose dysregulation may cause cancer [10]. Because these proteins are large and flexible, solving their structure remains difficult. Computational analyses of alpha-solenoids are therefore critical to model these proteins and better understand their biology and involvement in disease.

Analysis of protein sequence similarity is generally a good tool to discover many types of protein repeats. However, the extreme sequence divergence of alpha-solenoids limits the application of such methods to these repeats [11]. Accordingly, although the major domain databases (e.g. PFAM [12] and SMART [13]) contain profiles for repeats such as HEAT and Armadillo, their level of detection is poor. Methods of the prediction of these repeat types include iterative detection [14], [15] and Hidden Markov Model based searches of profiles derived from proteins of known structure [10].

Neural networks trained on positive examples have been successfully applied to detect structural motifs such as secondary structure and transmembrane helices [16] and therefore they offer an alternative to homology based detection of repeats. Following this idea, we previously developed a neural network (ARD, for Alpha-rod Repeat Detector, [9]) for the detection of alpha-solenoids. Here, we present an update of this method and of the predicted set of proteins known to be alpha-solenoids.

The resolution of novel protein structures of alpha-solenoids, some of them lacking sequence similarity to previously defined alpha-solenoid proteins, offered a chance to enhance the algorithm's training set and performance. We also improved the basic ARD algorithm to allow the detection of repeats with linkers of variable length between the two alpha helices of each repeat. After optimization of the parameters used to identify a protein as containing an alpha-solenoid by test on proteins of known structure from PDB, we could prove that the new algorithm (ARD2) has an improved coverage. The method is available as a public web tool at: http://cbdm.mdc-berlin.de/~ard2/.

The application of ARD2 on all available sequences from the TrEMBL database [17] allowed us to explore the distribution of alpha-solenoids across the tree of life. Our analysis suggests that alpha-solenoids have emerged through multiple events in Bacteria, Archaea and Eukarya, therefore constituting a case of convergent evolution. We also show that alpha-solenoids are highly represented in eukaryotic organisms, while in Prokaryota they are rare and concentrated in few taxa with a higher degree of compartmentalization than most prokaryotic species, such as Cyanobacteria [18], [19] or Planctomycetes [20].

## Results

### Detection of alpha-solenoid proteins using a neural network

To be able to detect alpha-solenoid regions more accurately and thus expand their definition, we improved an available detection tool based on a neural network, ARD (Alpha-rod Repeat Detection) [9]. Briefly, the neural network scores between 0 and 1 each of the amino acids of a protein sequence used as input. High scores identify the linker between the two alpha helices of a repeat unit. The optimization of the neural network is guided by the identification of high scoring hits in a sequence with appropriate periodicity evidencing the multiple repeat units of an alpha-solenoid. The neural network is trained with sets of positives in a supervised learning manner: thus, it is required to output a scoring value of one for the middle position of the linker of the repeat unit and a zero otherwise (see Methods for details).

**Figure 2:**
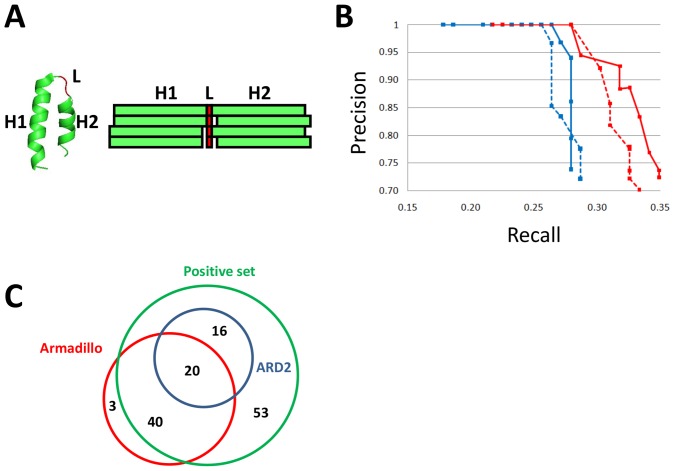
Detection of alpha-solenoid proteins using a neural network. (A) A repeat is made of two helices (H1 and H2) separated by a linker sequence (L). Two detection windows of 19 amino acids are considered, one for each helix. During detection, different window shifts are tested by sliding the input windows H1 or H2 one residue apart from the middle-residue (red box), as indicated by the gaps between red and green boxes. (B) Precision-recall curves comparing the performance of ARD2 in identifying alpha-solenoids in our PDB set using different sets of parameters. A protein was identified as containing an alpha-solenoid if it had 3 or more hits above a given score threshold spaced between 30 and 135 amino acids of each other. This restricts the hits to an expected periodic range within 30 to 40 amino acids. The blue discontinuous and continuous curves show performance for ARD and ARD2 training sets, respectively, without using window shifts. Discontinuous and continuous red curves show performance for ARD and ARD2 training sets, respectively, for a window shift of 1. Different points across each curve correspond to score thresholds from 0.80 to 0.90, with a 0.01 step. The best recall for a 100% precision is obtained when using the window shift and a score threshold of 0.87 (precision: 1.00, recall: 0.28). The ARD2 training set produced generally better results than the ARD training set, and resulted in the best value of precision × recall for a threshold score of 0.86 (precision  = 0.93, recall  = 0.32). (C) Comparison of structures recalled from the positive set (Table S1) by the Armadillo profile from InterPro and ARD2. Proteins detected outside of the positive set circle (Green) are consequently false positives (See Table S2 for a detailed list of the proteins detected).

To improve the detection of alpha-solenoids achieved by ARD [9] we changed the way hits are identified in that method. In ARD, the window of detection consists of 39 positions, 19 for the first helix, 19 for the second helix, and a middle linker of fixed length of 1 (Figure 2A). In ARD2, we allow the linker to have a variable length: we tested positions for the 19 amino acid windows at increasing distances from the middle position, which allowed detecting repeats with longer spacers between the helices.

In order to evaluate the performance of ARD2 we tested it in a redundancy-reduced set of 19,769 sequences of known structures (see Methods for details). Firstly, we identified 129 alpha-solenoids by examination of the results and literature analysis, which constitute our curated set of positives. Identification of any other sequence in the dataset as an alpha-solenoid is counted as a false positive. The list of PDB identifiers is available as Table S1. Other sequences with solved structures containing alpha-solenoids might exist but they will be very similar in sequence to our set and were therefore not considered.

At a 100% precision level a maximum recall of 0.28 was obtained if sequences were identified as containing alpha-solenoids if they had at least three hits with a minimum score of 0.86 and a distance between hits in the range of 30 to 135 residues (Figure 2B). This strict criterion was used hereafter for the automatic selection of candidates (Table S2). More relaxed criteria results in the identification of more proteins but with many false negatives. For example, using a 0.5 threshold in the score identifies 59 of the 129 positives (improving significantly the recall to 0.46), but also a total of 265 proteins of the 19,769 tested proteins were identified (that is, 206 false positives, precision is 0.22). For this reason we provide accessibility to the use of the method with a representation that allows studying the positions and scores of the hits found in a given sequence, without the application of any thresholds, to support detailed exploratory searches of individual sequences.

As mentioned above, a series of methods use profiles for the detection of various domains that form alpha-solenoids. Their coverage tends to be different from that of ARD2 as shown, for example, by a comparison of the ARD2 hits in our redundancy reduced PDB set of 19,769 sequences with the Armadillo domains reported in the InterPro sequence database [21] (Table S2; Figure 2C). This suggests that ARD2 can complement profile methods.

### Structural and functional properties of alpha-solenoids

Beyond their use in our benchmarking, the set of 129 curated positives gives insight into the structural and functional properties and context of alpha-solenoids. The distribution of functions in this set is dominated by the presence of karyopherins (26 proteins (20%) out of 129), although this observation might be biased by the tendency of researchers to solve structures of particular proteins. Karyopherins (alpha-importins alpha, beta-importins and transportins) are proteins that transport other proteins into and out of the nucleus. Other functions observed in the set of 129 alpha-solenoids include activation of transcription factors, protein biosynthesis, vesicle trafficking, DNA repair and RNA processing. We summarize some observations regarding the structure and ligand binding of these proteins, which challenge or expand our current knowledge on the function of alpha-solenoids.

#### Alpha-solenoids are not always part of the surface of proteins

**Figure 3:**
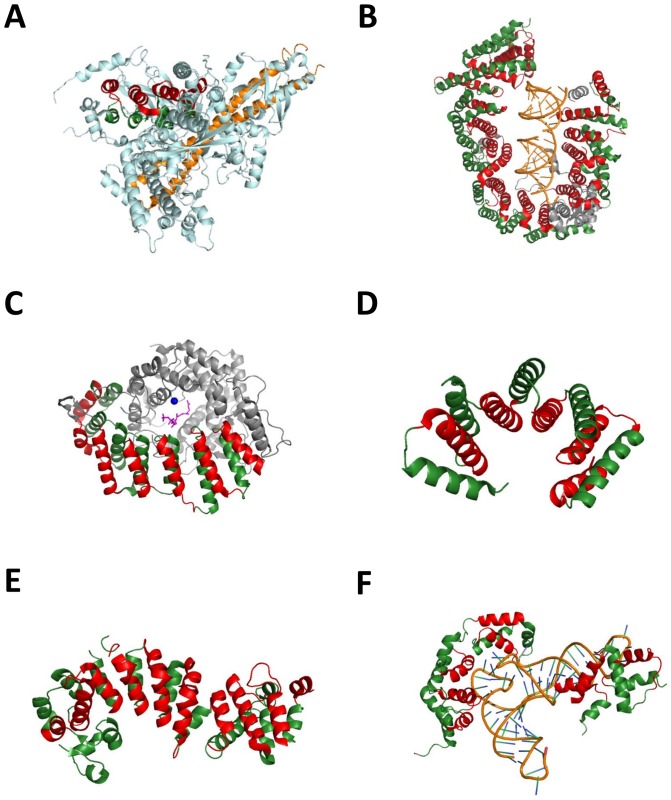
Examples of detected alpha-solenoid structures. Each repeat consists of two alpha-helices, depicted here in red and green. (A) HEAT repeats buried in the core of the PI3KC catalytic subunit p110alpha (cyan), in complex with p85alpha (orange) (PDB ID 3HHM [22]). (B) Alpha-solenoid binding RNA in exportin5 (PDB ID 3A6P [23]). (C) Lipid-binding protein. Isoprenoid lipid directly binding the HEAT repeats is colored in magenta, zinc atom in blue (PDB ID 3DRA [25]). (D) TPR repeats protein, virulence regulator from *Bacillus thuringiensis* (PDB ID 2QFC [26]). (E) Ankyrin repeats protein Q5ZSV0 from *Legionella pneumophila* (PDB ID 2AJA [57]). (F) Irregular alpha-solenoid, glutamyl-tRNA synthetase from *Thermotoga maritima* (PDB ID 3AL0 [61]).

Though alpha-solenoids usually form a surface for protein-protein interactions and are consequently located in the outer part of proteins, sometimes even shaping the complete structure of the protein, they can also be buried. Here, we present a structure with a previously unnoticed buried alpha-solenoid, p110alpha (Figure 3A, PDB ID 3HHM [22]), which is the catalytic subunit of the phosphatidylinositol 3-kinase alpha (PI3Kalpha) complex. The solenoid domain is involved in the docking of p85alpha, one of the proteins that help PI3Kalpha to scaffold properly. To our knowledge, this is the only solved structure displaying such an inner localization of an alpha-solenoid along with that of the homologous p110delta protein in mouse (PDB ID 2WXF).

#### Alpha-solenoids can interact with nucleic acids

Support for interaction with proteins is not the only function of alpha-solenoids. Exportin-5 is one example of alpha-solenoid interacting with nucleic acids (Figure 3B; PDB ID 3A6P; [23]). In this protein-RNA structure, exportin binds immature pre-microRNA in complex with RanGTP. The repeats form a big hairpin that wraps around the RNA. Interestingly, the authors of [23] point out that the RNA is only bound to the repeats via its backbone, which means that binding of exportin-5 to small RNA is independent of its nucleotide sequence. Exportin-5 both transports premature miRNAs from nucleus to cytoplasm and protects them from degradation by nucleases.

#### Alpha-solenoids can interact with lipids

We report two alpha-solenoid protein structures with bound lipids. Lipovitellin is mainly formed of an open cone of HEAT repeats inside of which lipids contact the protein (PDB ID 1LSH; [24]). To our knowledge, this is the first structure showing direct contact of an alpha-solenoid with lipids. The GGTase-I (geranylgeranyltransferase-I), is another alpha-solenoid that binds lipids. This protein catalyzes the fusion of lipids on proteins. Approximately half of its structure displays HEAT repeats (Figure 3C, PDB ID 3DRA [25]).

#### TPR repeats, ankyrin repeats and some irregular structures are identified as alpha-solenoids

The expansion of our method results in the novel identification of a wider set of alpha-solenoids that are non-homologous to HEAT or Armadillo repeats, stressing the fact that a sequence analysis method can detect multiple types of such structural elements, even if they are not related by statistically significant homology. The newly identified proteins include other repeats that are known to form alpha-solenoids and some much-distorted alpha-solenoids.

For example, we identified PlcR, the major virulence regulator from *Bacillus thuringiensis*, which is formed by TPR (Tetratricopeptide) repeats (Figure 3D, PDB ID 2QFC [26]), and an alpha-solenoid formed by ankyrin repeats from the *Legionella pneumophila* protein Q5ZSV0 of uncharacterized function (Figure 3E; PDB ID 2AJA). An example of very irregular alpha-solenoid is the bacterial glutamyl-tRNA synthetase (Figure 3F, PDB ID 3AL0; [27]), with repeats that are much more twisted respect to each other (about 90°) than in most alpha-solenoids.

To summarize, our survey expanded the definition of alpha-solenoids in terms of function (interaction with lipids and nucleotides), localization (presence in core of proteins possible), and morphology (TPR, ankyrin repeats and irregular alpha-solenoids can be detected). A summary of human alpha-solenoid structures is presented in Table 1.

**Table 1:** Functions of proteins with alpha-solenoids.

Function	Interaction	PDB ID	Protein name	Type	Reference
Protein transport	P/P	2JDQ	Importin subunit alpha-1	ARM	[63]
Protein transport	P/P	1IAL	Importin subunit alpha-2/pendulin	ARM	[64]
Protein transport	P/P	1IBR	Importin subunit beta-1/importin 90	HEAT	[29]
Protein transport	P/P	2OT8	Transportin-1 (Importin beta-2)	HEAT	[65]
Protein transport	P/P	1WA5	Re-exporter of importin subunit alpha	HEAT	[32]
TF coactivators	P/P	2Z6H	Catenin beta-1	ARM	[60]
TF coactivators	P/P	3OC3	Helicase MOT1	HEAT	[66]
Protein biosynthesis	P/N?	2IW3	Elongation factor 3A	HEAT	[67]
Protein biosynthesis	P/N	3AL0	Glutamyl-tRNA synthetase	HEAT	[61]
Enzyme scaffolding	P/P	2IAE	Protein Phosphatase PP2A subunit A	HEAT	[68]
Enzyme scaffolding	P/P	2PZI	Protein kinase PknG	HEAT	[69]
Enzyme scaffolding	P/P	2DQ6	Aminopeptidase N	HEAT	[27]
Enzyme scaffolding	P/P	3HHM	PI3Kalpha	HEAT	[22]
Substrate catalysis	P/P	2IAE	Protein Phosphatase PP2A subunit B	HEAT	[68]
Vesicle trafficking	P/P	1W63	AP1 Clathrin adaptor core	HEAT	[70]
Vesicle trafficking	P/P	2VGL	AP2 Clathrin adaptor core	HEAT/ARM	[71]
Vesicle trafficking	P/P	3GRL	p115 tether globular head domain	HEAT	[72]
Cytoskeleton	P/P	3OPB	She4p	HEAT	[73]
Cytoskeleton	P/P	2QK1	Protein STU2	HEAT	[74]
Ubiquitination/proteasome	P/P	1U6G	Cand1	HEAT	[31]
Ubiquitination/proteasome	P/P	1XQS	Hsp70-binding protein 1	ARM	[75]
Ubiquitination/proteasome	P/P	1VSY	Proteasome activator BLM10	HEAT	[76]
Ubiquitination/proteasome	P/P	3GAE	Ubiquitin fusion degradation 3	ARM	[77]
DNA damage	P/N	3JXY	Alkylpurine DNA glycosulase AlkD	HEAT	[78]
micro-RNA processing	P/N	3A6P	Exportin-5	HEAT	[23]
mRNA processing	P/P	3O2Q	Symplekin	HEAT/ARM	[79]
mRNA processing	P/P	1N52	Cap-binding protein	HEAT	[80]
mRNA processing	P/P	3D3M	Death associated protein 5 (DAP5)	HEAT	[81]
Lipid metabolism	-	3DRA	Geranylgeranyltransferase-I	HEAT	[25]
Lipid metabolism	P/L	1LSH	Lipovitellin	HEAT	[24]
Tumor suppressing	P/P	1UPK	Calcium-binding protein 39	ARM	[82]
Other function	P/P	2DB0	Hypothetical protein	HEAT	PDB
Other function	P/P	2AJA	Ankyrin repeat protein	ANK	PDB
Other function	P/P	2QFC	Virulence regulator	TPR	[26]
Other function	-	3LTJ	Artificial protein	HEAT	[83]
Other function	-	1LRV	Leucine-rich repeat protein	L-rich	[84]

Each protein is displayed with its PDB ID and the type of interaction its repeats are involved in. Though most of structures dock to proteins, we here point out the involvement of alpha-solenoids in protein-protein (P/P), protein-lipid (P/L) and protein-nucleic acid (P/N), either DNA or RNA. The diversity of function is broader than previously known.

### Functional analysis of human alpha-solenoids

Application of the ARD2 algorithm to the whole human proteome available in SwissProt (20,328 sequences; SwissProt version 15.6) indicated 99 alpha-solenoids (Table S3). We performed a Gene Ontology (GO) term and KEGG pathway enrichment analysis of these proteins, with the entire human genome as background (using DAVID; [28]). According to the identification of karyopherins, importins, exportins, and adaptins, we observed a significant enrichment in GO functions and subcellular locations related to several of these proteins such as “Protein transporter activity” (p-value  = 4.9e-39; Benjamini-Hochberg corrected), “Intracellular trafficking and secretion” (p-value  = 8.6e-21), “nuclear pore” (p-value  = 1.2e-17), “Nuclear localization sequence binding” (p-value  = 1.3e-9), and “coated membrane” (p-value  = 1.3e-12).

This analysis indicated a tendency of alpha-solenoids to interact with many protein partners according to the current experimental information on human and yeast PPIs. This result is in agreement with many of the functions observed for alpha-solenoids, which often require protein-protein interactions.

It was proposed previously by us and others [1], [7] that alpha-solenoids are involved in protein-protein interaction, and indeed there are several solved structures of complexes showing the interaction of alpha-solenoids with proteins. Examples are the complexes of the alpha-solenoid of beta-importin with Ran (PDB:1IBR; [29]), of the alpha-solenoid of alpha-importin with the NLS (nuclear localization signal) peptide from c-myc (PDB:1EE4; [30]), of the alpha-solenoid of Cand1 (TIP120) with Cul1 (cullin 1) (PDB:1U6G; [31]), and of the alpha-solenoid of exportin CSE1P with both KAP60P and RanGTP (PDB:1WA5; [32]).

**Figure 4:**
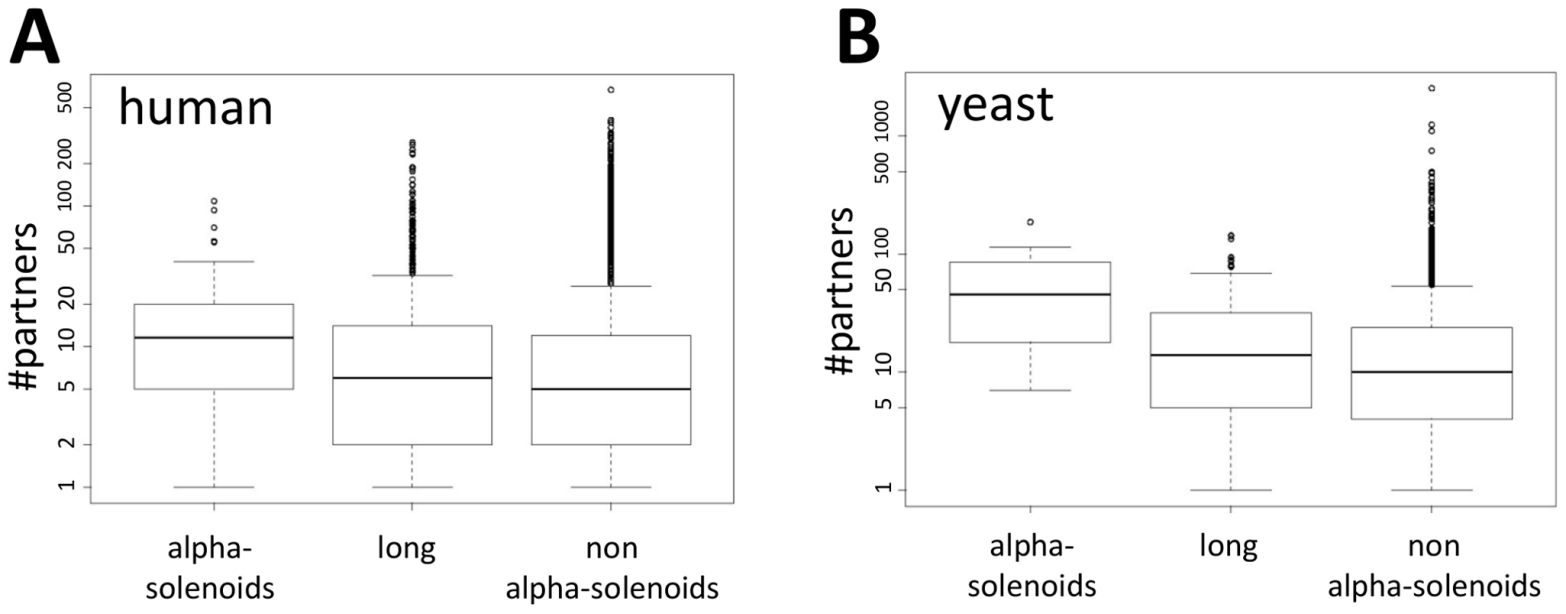
Proteins with alpha-solenoids establish more protein interactions than other proteins. Each box-plot indicates the distribution of interacting partners for proteins predicted to contain alpha-solenoids, long proteins not predicted to contain alpha-solenoids, and all proteins not predicted as containing alpha-solenoids. (A) Human proteins. (B) *Saccharomyces cerevisiae* proteins. Boxes represent the values between first and third quartile of the distributions. The horizontal line inside of the boxes indicates the median value. Circles indicate the outliers. All pairwise differences are significant (see text for details).

In order to support the hypothesis that protein interactions are a major function of alpha-solenoids, we examined the experimental information about PPIs in the literature as collected in the HIPPIE database [33], [34]. We observed that human proteins predicted to contain alpha-solenoids have significantly more interaction partners than proteins not having them (p-value <1.3e-06; Figure 4A). Considering that some protein features, such as alpha-solenoids, can be more easily found in long proteins and that these have more interactions, we also compared to the number of partners in proteins longer than the average predicted alpha-solenoid protein (1,086 aa versus an average of 553 aa for human proteins). The difference was also substantial (p-value <8e-04; Figure 4A). Similar results were obtained for the PPIs of the yeast *Saccharomyces cerevisiae* (as defined in BioGrid [35]): alpha-solenoid predicted proteins had significantly more interactors than non-alpha solenoid (p-values <7e-9 and <5e-6, respectively; Figure 4B).

### Human proteins newly identified to contain alpha-solenoids

ARD2 increases the count of human proteins predicted to contain alpha-solenoids with respect to ARD from 89 to 99 (Table S3). Here we describe six human proteins newly identified as alpha-solenoids: LRRK2, RTTN, TRIP12, UNC45, DNAJC13 and IFRD1 (Table 2). Sequence similarity analyses indicate that, as with most eukaryotic alpha-solenoids, they have homologs only within Eukarya, being mostly conserved within Chordata. All six were confirmed by InterPro scan (ARM like), although the predictions did not completely agree positionally.

**Table 2:** New human proteins with alpha-solenoids.

Name (SwissProt accession number)	Function	Conservation^1^	ARD2	InterPro ARM
**LRRK2. Leucine-rich repeat serine/threonine-protein kinase (Q5S007)**	serine/threonine kinase	Dm Bf Ci	360; 408; 452; 494	163–619
**RTTN. Rotatin (Q86VV8)**	Axial rotation, left-right specification of body	Dm Bf Ci	1305; 1377; 1425;	1–954, 1422–1445, 1602–1691, 1846–1956, 2017–2225
**TRIP12. E3 ubiquitin-protein ligase (Q14669)**	Ubiquitination	At Sc Dm Bf Ci	491; 532; 613	357–379, 436–938
**UNC45A (Q9H3U1)**	Co-chaperone of Hsp90, cell proliferation, muscle cell development, possible cytoskeletal function	Dm Bf Ci	448; 488; 537	89–350, 403–932
**DNAJC13. Required for receptor-mediated endocytosis 8 (O75165)**	Co-chaperone of Hsc70, receptor mediator endocytosis	At Dm Bf Ci	1783; 1826; 1865	445–1968, 1988–2191
**IFRD1. Interferon-related developmental regulator 1 (O00458)**	Embryotic development, muscle development	At Sc Dm Bf Ci	93; 136; 176	84–326

1 Orthologs were searched for in Sc: *Saccharomyces cerevisiae*, At: *Arabidopsis thaliana*, Dm: *Drosophila melanogaster*, Bf: *Branchiostoma floridae*, Ci: *Ciona intestinalis*.

**Figure 5:**
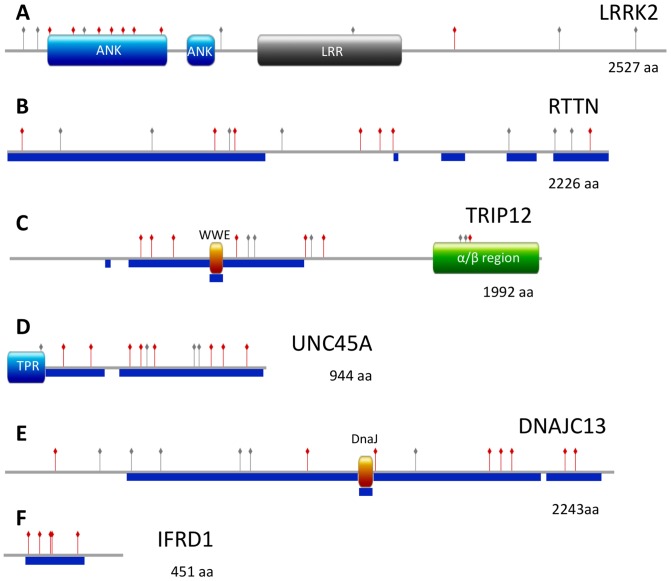
Domain organization of six predicted alpha-solenoid proteins. Alpha-solenoid repeat units predicted by ARD2 are displayed with red needles (score of or above 0.87). Other scores above threshold 0.30 are represented with grey needles. For comparison, Armadillo regions predicted by InterPro are displayed as blue boxes. Other predicted domains are displayed with labels. (A) LRRK2, (B) RTTN (rotatin), (C) TRIP12, (D) UNC45A, (E) DNAJC13, and (F) IFRD1.

LRRK2 (Figure 5A) is a large (2527 aa) serine/threonine protein kinase whose mutation can cause Parkinsonism [36], and is predicted by ARD2 to have repeats in the region 360–494. The periodicity of the repeats was coherent with secondary structure predictions (using Jpred3; [37]). A recent sequence analysis studied other structural domains in this protein: an ankyrin repeat domain (an alpha-solenoid), a WD40 domain (a barrel of beta-sheet repeats), and a leucine rich repeat (LRR) domain (solenoid alpha-beta repeats) [38]. This detailed analysis concluded that the ARM domain contains 13 repeats at positions 49–657. Including low score predictions, ARD2 covers 10 of those repeats (Figure 2A). This suggests that it is useful to examine sub-optimal ARD2 matches.

**Figure 6:**
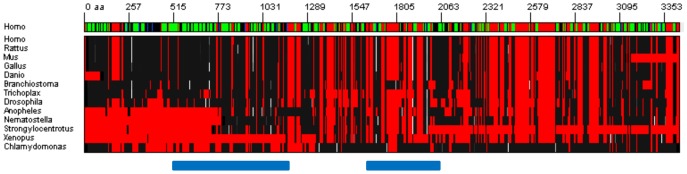
Alignment of rotatin homologs. A multiple sequence alignment of human rotatin and homologs in other species was produced and represented using BiasViz [62]. Top lane: Jpred3 2D prediction for human rotatin (red: gaps, green: alpha-helix, blue: beta-strand). Bottom part: multiple sequence alignment (red: gaps, black to white: score of ARD2 prediction from 0 to 1). Most of the secondary structure prediction is alpha-helical. Clusters of periodic alpha-solenoid hits can be seen at the positions indicated by the blue bars. Other scattered hits are distributed through the entire alignment.

RTTN (Rotatin; Figure 5B) is a large protein (2226 aa) involved in axial rotation and left-right specification of the body [39]. Similarly to its ortholog in *Drosophila* (Ana3), this protein interacts with the mitotic centrosomes and is required for cilia function [40]. ARD2 most significant hits fall in the 1300–1450 region but other less significant hits from ARD2 and InterPro suggest that the whole protein might be composed of alpha-solenoids. To support this hypothesis we collected distant homologs of this protein, including green algae *Chlamydomonas reinhardtii*, and observed the wide distribution of the ARD2 hits in members of this family, suggesting that the entire protein is composed of alpha solenoids (Figure 6).

TRIP12 (Figure 5C) is an E3 ubiquitin-protein ligase [41]. A previous sequence analysis of this large protein (1992 aa) suggested two regions of HEAT repeats interspersed by an ADP-ribose binding module termed WWE (at positions 749–798 aa) [42]. ARD2 matches both HEAT repeat regions with good scoring hits. The InterPro ARM prediction overlaps the small WWE domain, whose structure is mostly composed of beta-strands [43]. No ARD2 hits were obtained in the region.

UNC45A (Figure 5D) is a co-chaperon of Hsp90 involved in the correct folding of myosin during development [44]; its ortholog in *Drosophila* is a key protein for the development and function of the heart [45]. ARD2 and InterPro ARM-like hits covered large portions of the sequence but for an N-terminal region that forms tetratricopeptide repeats (TPR) (residues 1-135; PDB:2DBA; unpublished, RIKEN structural genomics initiative). Predictions are similar for the close paralog UNC45B (not shown).

DNAJC13 (aka RME8) (Figure 5E) is a large (2243 aa) co-chaperon of Hsc70 required for receptor-mediated endocytosis [46]. Both ARD2 and InterPro ARM-like hits cover most of the sequence, although the ARD2 hits spare a predicted DnaJ or J domain (at positions 1301–1354) that is however wrongly included in the InterPro ARM prediction.

IFRD1 (Figure 5F) is an interferon-related protein, involved in muscle development and also related to cystic fibrosis lung disease [47]. Highly scoring ARD2 hits suggest a central alpha-solenoid in this protein. Similar results are obtained for the close human paralog IFRD2 (not shown).

### Global survey of alpha-solenoids

**Figure 7:**
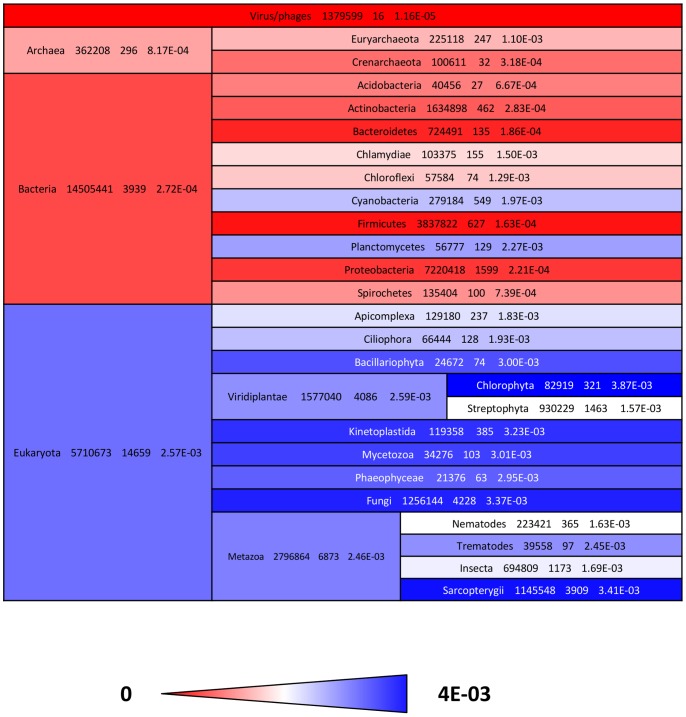
Alpha-solenoids in complete genomes. Fraction of alpha-solenoids in proteins from 31 taxonomic divisions.

In order to get functional and evolutionary information about alpha-solenoids, we used ARD2 with all protein sequences available on the TrEMBL database (22 million sequences; release 2012_05) and found a total set of 18,910 alpha-solenoids. We examined their distribution across the tree of life by calculating the percentages of alpha-solenoids for proteins from species arranged in 31 major taxonomic divisions (Figure 7).

Regarding the major domains of life, the observed frequency of alpha-solenoids was the lowest in virus (1.2e-5), higher in Bacteria (2.7e-4) and Archaea (8.2e-4), and the highest in Eukaryota (2.6e-3). The eukaryotic taxa analysed reflected relatively homogeneous values with most taxa having frequencies above 2.0e-3. In contrast, in Prokaryota, Cyanobacteria and Planctomycetes stand out with values of 2.0 e-3 and 2.3e-3, respectively, which are comparable to the average value for eukaryotes.

In Cyanobacteria, a large fraction of alpha-solenoids detected contain HEAT PBS domains (for Phycobilisome, a complex of molecules participating in light harvesting). This domain has been predicted to form alpha-solenoids and, accordingly, it is detected by ARD2. For example, in cyanobacteria *Nostoc punctiforme*, 15 of the 17 detected alpha-solenoids contain the HEAT PBS domain. This suggests that alpha-solenoids in Cyanobacteria correspond to gene duplications of a protein family involved in photosynthesis. Such HEAT PBS domains are found in the three main groups of Cyanobacteria (Chroococcales, Nostocales and Stigonematales) suggesting the concomitant emergency of this family of alpha-solenoids and Cyanobacteria about 3.5 billion years ago.

In Planctomycetes we observed a wider distribution of alpha-solenoid families than in Cyanobacteria. For example, in Planctomycetes *Blastopirellula marina DSM 3645*, the largest family we identified among the 21 detected alpha-solenoids had 9 sequences (of uncharacterized function). We observed that in *Rhodopirellula baltica*, for instance, HEAT PBS proteins constitute only 2 of the 13 alpha-solenoids detected. Presence of PBS lyase related-sequences was surprising, as Planctomycetes do not harvest light.

Regarding Archaea, the majority of sequences identified were homologous to bacterial sequences, in particular those containing the HEAT PBS domain, with few (less than 30%) Archaeal specific families. For example, Euryarcheota *Methanoculleus marisnigri*, isolated from anaerobic digestors and aquatic sediments, has PBS domains in 9 out of the 10 alpha-solenoids detected. Regarding the function of these proteins, HEAT PBS domain containing OE2401F protein from *Halobacterium salinarum*, a halophilic marine Euryarcheota, was found to be associated to flagella [48]. Its closest homolog in Bacteria are from Cyanobacteria (matches spanning the entire amino acid sequence with a level of 28% identical residues), suggesting that some HEAT PBS proteins in Cyanobacteria might have a motility function.

In all the set of viral proteins we identified just 16 alpha-solenoids. We detected clear homologs in their own hosts for 7 of them (Chlorella or Streptococcus, homologous proteins having at least 60% of identity on 75% of their length). Some of the remaining 9 sequences belong to human hepatitis C virus, showing homology to sequences in Eukaryota (for example, the translation elongation factor 3 from *Phaeocystis globosa virus 14T*; UniProt G8DER4). Consequently, these viral sequences seem to be the result of horizontal transfer.

## Discussion

We have generated an improved method for annotation of alpha-solenoids and an updated collection of identified alpha-solenoids.

The examination of recently published protein structures identified to contain alpha-solenoids allowed us to extend the number of functions covered by this fold. At the structural level we were able to show that the interaction function of alpha-solenoids extends beyond proteins to interactions with DNA, RNA and lipids. Moreover, the existence of structurally buried alpha-solenoids (such as those that we identified in PI3Kalpha) suggests also their participation in the formation of protein cores. The protein-protein interaction function of alpha-solenoids, remain however as its most general feature: the analysis of the distribution of alpha-solenoids in the human and yeast PPI network indicated that, in general, alpha-solenoid containing proteins have more interacting partners compared to proteins of similar length not having them.

We presented then the first analysis of the distribution of alpha-solenoid proteins in the tree of life. They are found to be generally rarer in non-eukaryotic organisms than in eukaryotic ones. While in Eukarya around one in 400 proteins contains alpha-solenoids, this frequency is one order of magnitude lower in Bacteria (many species have none), and Archaea are in between with a frequency of one in around 1200 proteins.

We interpret the lack of significant sequence similarity between several families of alpha-solenoids (e.g. prokaryotic PBS lyase repeats and eukaryotic alpha-solenoids) and the phylogenetic distributions of such families as indicating that several bacterial and eukaryotic alpha-solenoid protein families have emerged independently. Some functions associated to these proteins support this: for example, prokaryotic PBS lyase repeats have associated functions that are not seen in Eukaryotic alpha-solenoids, such as photosynthesis in Cyanobacteria. This agrees with the previous suggestion derived from structural studies that alpha-solenoid repeats are relatively cheap to evolve [15].

Eukaryotic cells are more complex than prokaryotic cells, with presence of a nucleus and organelles. Therefore the former need more protein transport and protein interactions than the latter for the management of those organelles and the trafficking of material within them. Alpha-solenoids are useful as scaffolds for protein interaction and could facilitate the increase of protein cross-talk and hence of cellular complexity. On the other hand, alpha-solenoids are elongated and flexible and it may be difficult to fold them properly, therefore requiring a developed machinery to fold proteins and keep them from aggregating, which only appeared late in eukaryotic evolution.

This would explain why repeats forming elongated solenoids are mostly Eukaryotic specific. For example, Pfam and SMART place about 98% of Armadillo repeats in Eukaryota. Several other rod-forming repeats such as alpha-helical ankyrin (SMART: 87%; Pfam: 75%) and HAT (SMART: 97%; Pfam: 100%), and alpha-beta Leucine rich repeats (SMART: 93%) follow a similar trend. Alpha-helical TPR repeats are an exception and are highly represented in Bacteria (SMART: 55%), but this could be due to the fact that they display fewer repeats (generally three) and are therefore easier to handle than longer solenoids.

**Figure 8:**
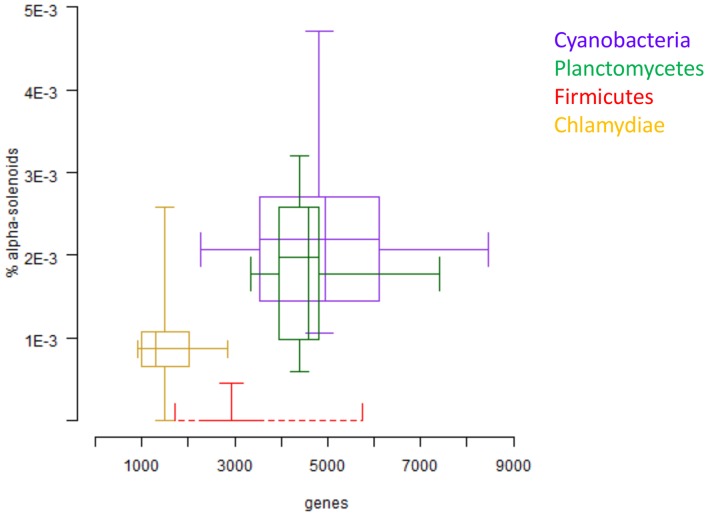
Percentage of alpha-solenoids versus number of genes. Two-dimensional box plot of percentage of alpha-solenoids against genome size averaged for several representative species with completely sequenced genomes from four bacterial groups: Cyanobacteria, Planctomycetes, Firmicutes and Chlamydiae. Each box shows the distribution of one of these four groups and summarizes two distributions: the percentage of alpha-solenoids associated to the genome of species of that group in the vertical direction, and the size of the genomes of the species of that group in horizontal direction. In each direction, the box is limited by first and third quartile of the distributions. The middle line (horizontal or vertical) inside of the boxes indicates the median value.

Interestingly, some particular prokaryotic taxa such as Cyanobacteria and Planctomycetes have fractions of alpha-solenoids comparable to Eukaryota. Both Cyanobacteria and Planctomycetes have a fairly large genome size in comparison to other prokaryotic species [49], and are the only known bacterial groups to possess morphological complexity [50] and their own cell compartments [18], [51], [52]. Comparison to other bacterial groups such as Firmicutes and Chlamydiae shows that larger genome sizes correspond to a larger fraction of alpha-solenoids (Figure 8). Presence of chaperones in both taxa [53], [54] shows that they have the molecular machinery to support the emergence of alpha-solenoids. In conclusion, the increased percentage of alpha-solenoids in organisms with larger genomes could be due to two reasons, which do not exclude each other. Firstly, alpha-solenoids could facilitate the emergence of large cellular machineries because of their elastic and interaction properties; secondly, the proper folding of complex protein structures such as alpha-solenoids by itself requires advanced folding machinery.

Thanks to the current expansion of the method of detection we are starting to detect some other types of alpha-solenoids beyond those composed of Armadillo and HEAT repeats, such as ankyrin repeats, and TPRs. This does not mean that they have sequence homology to Armadillo and HEAT repeats, or that their profiles need to be revised, but that detection of alpha-solenoids would be identifying sequences adopting a similar fold that would have been reached by convergent evolution from different evolutionary paths. The mechanical properties of ankyrin repeats have been discussed [55] and approach those of HEAT repeats [11]. We hope that with the resolution of novel structures we might be able to expand even further the detection of alpha-solenoids and unify their detection. We must note however that the method we use is extremely sensitive to the choice of sequences used in the training dataset. An important point was to have a large number of structures to verify the performance of the method, which resulted in an increase in the coverage including the detection of novel alpha-solenoids in human sequences and in sequences from other species.

## Methods

### Neural network for repeat detection

ARD2 is a tool for detection of alpha-solenoids that uses a neural network trained with a set of canonical sequences with alpha-solenoid repeats to later detect these motifs on query sequences. It is based on the same formalism as our previously developed tool ARD [9]. Briefly, the neural network has three layers of neurons with non-linear sigmoid activation function: an input layer of 39 times 20 neurons, where 39 is the number of sequence positions scanned and 20 the number of possible amino acids, a hidden layer of 3 neurons, and an output layer of one neuron. The neural network is trained to identify the central position of an alpha-solenoid repeat using the back-propagation algorithm. More details about the neural network architecture optimization and training can be found in the supplementary material of [9].

While the neural network fundamental parameters remain those used for ARD, here, we modified the use of the neural network algorithm to identify alpha-solenoids, to make it less restrictive than in ARD. Previously, repeats were detected using a 39-residue window expecting to detect a first alpha-helix, a central residue expected to be in coil structure, followed by a second alpha-helix. In reality, alpha-solenoid repeats show variable spacing between their two helices. In order to detect more repeats, we allowed the central linker to have a length greater than 1. For each position tested as central residue of a repeat, we not only tested the immediate 19 neighboring residues on both sides of the central residue but also examined as alternatives the 19 residues neighbor to position −1 and +1 from the central residue. This window shifts now allow the linker to be 1 to 3 amino acids long (Figure 2A). Therefore, for each central position tested four combinations of window displacements are tested and the maximum score obtained and corresponding window displacements are reported.

Therefore, we evaluated the identification of alpha-solenoids considering variations in four elements: the score threshold (a real value between 0 and 1), using the window shift described above or not, the distance between positions with scores above the threshold to be accepted as hits, and finally the minimal number of repeats to be detected in sequence.

As training set for the identification of individual repeats we started with 27 HEAT repeat containing sequences determined from a high quality alignment [56]. We then tested the expansion of the training set with sequences from our set of alpha-solenoids with known structures (Table S1). The algorithm was very sensitive to changes of the training set. The addition of an Ankyrin protein (2AJA [57]) allowed an improvement of the results (Figure 2B). The final training set of 28 proteins is available as Table S4.

To optimize the algorithm of alpha-solenoid detection, we applied it to protein sequences with structures in the Protein Data Bank (see below). The results were validated by mapping the ARD2 hits on the corresponding PDB structure for visual inspection using PDBpaint [58]. Positives were used to determine the precision and recall of each combination of parameters and training datasets.

We selected the combination that had the best recall for a precision of 100% (best results are shown on Figure 2B). The best performance was observed for a recall of 0.28. The parameters used were the following: a minimum of 3 repeats separated by a distance in the range [30,135], and a threshold of 0.87.

The method was able to identify sequences as alpha-solenoids that had no significant sequence similarity to any of the 28 sequences used in the training set. For example, the E-values of sequence similarity (according to BLAST) to the best match to the sequences in the training dataset were above 0.01 for human rotatin (UniProt ID: Q86VV8) (E-value  = 0.071) and for predicted proteins UniProt ID: Q7ULY0 (from *Rhodopirellula baltica*, E-value  = 0.16) and UniProt ID: A8JFV2 (from *Chlamydomonas reinhardtii*, E-value  = 0.047).

Since the method of identification of alpha-solenoids relies on finding enough repeats at expected distances, such identification works better with alpha-solenoids without insertions. In any case, the web tool offers the scores of detection of individual repeats, which are not filtered by score thresholds or by the distances between the hits found.

### Datasets of protein sequences

For the optimization of the detection of alpha-solenoids by application of the trained neural network we obtained sequences of proteins of solved structure from the Protein Data Bank [57]. A total of 174,488 protein sequences were classified into 23,710 clusters using a conservative algorithm [59]. After removing sequences shorter than 20 amino acids and those whose PDB structure had no acceptable quality according to the NCBI standard (defined in the nrpdb.latest file; ftp://ftp.ncbi.nih.gov/mmdb/nrtable/nrpdb.latest) 19,769 clusters remained. For each cluster, we selected the best PDB structure according to the following parameters, in decreasing order of importance: best resolution of solved structure, lowest percentage of unknown residues, lowest percentage of missing residues, longest sequence.

### Statistical analysis of protein-protein interactions

Protein-protein interactions were retrieved from the HIPPIE database [34]. Comparison of average number of interaction partners between alpha-solenoid proteins and other proteins, as well as comparison of alpha-solenoid proteins and long proteins, were performed using Wilcoxon–Mann–Whitney tests.

## Supporting Information

Table S1 **Positive set of PDB structures with alpha-solenoids.**
(XLS)

Table S2 **Comparison of performances for ARM profile and ARD2.**
(XLS)

Table S3 **Human protein sequences from SwissProt predicted to contain alpha-solenoids by ARD2.** MC stands for Manual Classification.
(XLS)

Table S4 **Training set of ARD2.**
(XLS)
